# Neonatal mortality attributable to preterm births associated with maternal HIV infection in sub-Saharan Africa

**DOI:** 10.1097/QAD.0000000000004463

**Published:** 2026-02-11

**Authors:** Nkazi Nchinda, Joris Hemelaar

**Affiliations:** Infectious Disease Epidemiology Unit, Nuffield Department of Population Health, University of Oxford, Oxford, UK.

**Keywords:** HIV, human immunodeficiency virus, modelling, neonatal death, preterm birth, sub-Saharan Africa

## Abstract

**Objectives:**

We aimed to estimate the burden of neonatal mortality attributable to preterm births associated with maternal HIV infection in sub-Saharan Africa in 1990–2020.

**Design:**

Modelling study.

**Methods:**

Estimates of the excess risk of preterm birth among pregnant women with HIV (WWH) in sub-Saharan Africa in 1990–2020 were obtained from a systematic review and meta-analysis. Data on preterm birth, neonatal mortality, and deaths <1 year directly caused by HIV/AIDS were obtained from the Global Burden of Disease Study 2021. Information on antiretroviral treatment received by WWH in sub-Saharan Africa in 1990–2020 were obtained from UNAIDS. These data were used to estimate neonatal mortality due to HIV-attributable preterm birth in countries in sub-Saharan Africa in 1990–2020.

**Results:**

In 1990–2020, there were an estimated 85 288 (95% confidence interval 53 147–136 921) neonatal deaths due to HIV-attributable preterm births in sub-Saharan Africa. The number of neonatal deaths due to HIV-attributable preterm births in sub-Saharan Africa increased during the 1990s, before decreasing in the 2000s, reaching a nadir around 2012, and increasing again within the last decade to levels in 2020 that were similar to their highest levels in the early 2000s. In 2020, neonatal deaths from HIV-attributable preterm birth exceeded direct HIV/AIDS infant deaths <1 year in sub-Saharan Africa, and by 5–25-fold in countries with a high HIV prevalence.

**Conclusions:**

Neonatal deaths due to HIV-attributable preterm birth are increasing and now exceed direct HIV/AIDS infant deaths <1 year in sub-Saharan Africa, particularly in countries with a high HIV prevalence.

## Introduction

Neonatal morbidity and mortality were the leading causes of disability adjusted life years worldwide in 2020 [1]. Globally, preterm birth is the most important cause of neonatal and child mortality [2,3], with an estimated 13.4 million cases in 2020, with no change in the global preterm birth rate since 2010 [4]. Sub-Saharan Africa has the highest rates of neonatal and child mortality globally [5].

In 2024, 15.7 million women of childbearing age lived with HIV globally, with the vast majority living in sub-Saharan Africa [6]. Since 2015, the World Health Organization (WHO) has recommended immediate, lifelong antiretroviral therapy (ART) for all pregnant women with HIV (WWH), as it improves their health and prevents vertical transmission of HIV [7]. Recent evidence indicates that both maternal HIV infection and ART affect the risk of adverse perinatal outcomes, including preterm birth. Untreated pregnant WWH have an increased risk of preterm birth relative to HIV-negative women [8]. Notably, while WWH treated with zidovudine (ZDV) monotherapy historically had a risk of preterm birth comparable to HIV-negative women, the same protective benefit does not appear to extend to ART regimens [9–11]. Consequently, HIV and ART were associated with almost 2 million preterm births in sub-Saharan Africa in 1990–2020 [12].

Although preterm birth is the leading cause of neonatal mortality globally, the impact of preterm births associated with maternal HIV infection and ART on neonatal mortality in sub-Saharan Africa remains unknown. To fill this evidence gap, we estimated the neonatal mortality due to HIV-attributable preterm births in sub-Saharan Africa in 1990–2020.

## Methods

### Preterm birth and neonatal mortality data

Data on preterm birth and neonatal mortality in countries in sub-Saharan Africa during 1990–2020 were extracted from the Global Burden of Disease (GBD) Study 2021 [1]. The general methodological basis of the GBD studies is described elsewhere [13]. For all countries in sub-Saharan Africa, the number of preterm births, number of neonatal deaths (<28 days of life) due to preterm births, and number of neonatal deaths from all causes were extracted, including 95% confidence intervals (CIs). Data on deaths <1 year directly caused by HIV/AIDS (with 95% CIs) were also extracted, as estimates for neonatal deaths due to HIV/AIDS were not available.

### HIV-attributable preterm births

A systematic review and meta-analysis provided estimates for the unadjusted excess risk of preterm birth (with 95% CIs) of pregnant WWH in sub-Saharan Africa relative to HIV-negative women [12]. This meta-analysis leveraged a large data set derived from 34 cohort studies including 399 558 women in 14 countries in sub-Saharan Africa [12]. Data on the number of pregnant WWH, HIV prevalence among pregnant women, and antiretroviral treatment received by pregnant WWH in 1990–2020 were obtained from UNAIDS [14]. Pregnant WWH were analyzed according to four categories based on their exposure to ART: no ART, ZDV monotherapy, antenatal ART, and preconception ART. ART was defined as receiving any combination of three or more antiretroviral drugs. The excess risk of preterm birth was estimated separately for each of the four categories of WWH, relative to women without HIV. The numbers of WWH in each treatment category in each country in 1990–2020 and the estimated excess risks of preterm birth associated with each category were used to estimate the number of preterm birth events for each country and each year. Excess preterm birth events among WWH compared to HIV-negative women were defined as HIV-attributable preterm births. Preterm birth was defined as birth <37 weeks of gestation. The term WWH was used for consistency with the databases used for this analysis, though not every pregnant person is a woman.

### Statistical analysis

Neonatal deaths from HIV-attributable preterm birth were estimated by multiplying the proportion of all preterm births attributable to HIV by the preterm birth-specific neonatal mortality rate, then applying this product to total neonatal deaths. We performed a 25 000 sample Monte Carlo simulation to estimate 95% CIs of derived estimates. Djibouti and Somalia were excluded due to insufficient data on excess preterm birth attributable to HIV. Mauritius was excluded due to a lack of necessary GBD estimates. Calculations were performed using R software (v4.5.1).

### Role of the funding source

This study did not receive any funding. The corresponding author had full access to all the data in the study and had final responsibility for the decision to submit for publication.

## Results

### Neonatal mortality associated with HIV-attributable preterm births in sub-Saharan Africa in 1990–2020

Data were available for a total of 44 countries in sub-Saharan Africa (Table 1). In 2020, HIV-attributable preterm births led to an estimated 2677 (95% CI 1643–4347) neonatal deaths (Table 1). HIV-attributable preterm births were associated with 85 288 (53 147–136 921) neonatal deaths in sub-Saharan Africa in 1990–2020, with the highest number of neonatal deaths due to HIV-attributable preterm birth in South Africa (13 411 [8865–20 261]) (Table 1).

**Table 1:** Neonatal mortality associated with HIV-attributable preterm birth and direct HIV/AIDS deaths in 1990–2020.

Country	Proportion of neonatal deaths due to HIV-attributable PTB (%; 2020)	Number of neonatal deaths due to HIV-attributable PTB (2020)	Number of neonatal deaths due to HIV-attributable PTB (1990–2020)	Number of deaths <1 year directly due to HIV/AIDS (2020)	Number of deaths <1 year directly due to HIV/AIDS (1990–2020)	Neonatal deaths due to HIV-attributable PTB / Direct HIV/AIDS deaths <1 year (ratio; 2020)
Angola	0.19 (0.11–0.33)	46 (27–79)	1292 (752–2215)	199 (187–208)	3819 (3574–4075)	0.23 (0.13–0.40)
Benin	0.08 (0.05–0.14)	12 (7–21)	576 (341–969)	6 (5–7)	1078 (1004–1145)	2.12 (1.25–3.56)
Botswana	2.31 (1.32–4.00)	22 (14–36)	578 (354–943)	1 (1–1)	1370 (1209–1547)	27.61 (16.52–45.83)
Burkina Faso	0.09 (0.05–0.16)	25 (15–41)	1158 (696–1923)	13 (12–13)	3439 (3268–3601)	1.92 (1.15–3.21)
Burundi	0.10 (0.05–0.17)	11 (7–19)	850 (519–1396)	6 (6–7)	3386 (3216–3527)	1.73 (1.02–2.95)
Cameroon	0.31 (0.18–0.53)	75 (45–124)	2391 (1432–3991)	94 (88–100)	8394 (7794–8976)	0.80 (0.48–1.33)
Cape Verde	0.06 (0.03–0.11)	0 (0–0)	5 (3–8)	0 (0–0)	27 (25–29)	NA
Central African Republic	0.12 (0.07–0.21)	9 (5–15)	554 (328–939)	19 (18–21)	3680 (3380–3976)	0.45 (0.26–0.77)
Chad	0.05 (0.03–0.10)	15 (9–26)	672 (398–1134)	13 (12–13)	1861 (1791–1935)	1.17 (0.69–1.99)
Comoros	0.00 (0.00–0.01)	0 (0–0)	0 (0–1)	0 (0–0)	1 (0–1)	NA
Congo	0.49 (0.28–0.88)	11 (6–18)	353 (210–594)	24 (22–26)	2066 (1796–2322)	0.44 (0.26–0.77)
Côte d’Ivoire	0.11 (0.07–0.19)	32 (19–53)	2300 (1411–3745)	48 (45–50)	13261 (12322–14160)	0.67 (0.41–1.11)
Democratic Republic of the Congo	0.10 (0.06–0.18)	68 (40–114)	2893 (1621–5166)	18 (17–19)	14022 (13282–14685)	3.66 (2.15–6.20)
Equatorial Guinea	0.79 (0.40–1.56)	5 (3–10)	78 (45–138)	16 (15–17)	439 (385–492)	0.34 (0.18–0.63)
Eritrea	0.01 (0.01–0.02)	0 (0–1)	29 (17–50)	8 (7–8)	611 (567–653)	0.05 (0.03–0.08)
Eswatini	5.37 (3.07–9.32)	25 (15–41)	683 (410–1138)	9 (9–9)	4135 (3935–4332)	2.76 (1.66–4.57)
Ethiopia	0.03 (0.02–0.06)	30 (19–49)	2919 (1885–4517)	52 (50–54)	10057 (9545–10512)	0.58 (0.36–0.94)
Gabon	0.40 (0.21–0.75)	3 (2–5)	166 (98–282)	4 (4–5)	612 (531–693)	0.68 (0.38–1.22)
Gambia	0.08 (0.05–0.16)	1 (1–3)	42 (25–71)	5 (4–5)	219 (196–242)	0.31 (0.17–0.55)
Ghana	0.10 (0.05–0.20)	20 (11–39)	960 (523–1769)	31 (29–32)	4438 (4035–4858)	0.67 (0.34–1.29)
Guinea	0.04 (0.02–0.08)	7 (4–12)	405 (243–676)	12 (11–13)	1186 (1119–1245)	0.57 (0.32–1.00)
Guinea-Bissau	0.17 (0.09–0.30)	3 (2–6)	147 (88–245)	14 (13–15)	306 (284–328)	0.24 (0.14–0.42)
Kenya	0.35 (0.22–0.58)	76 (49–119)	4143 (2603–6594)	143 (137–149)	35918 (34248–37485)	0.53 (0.34–0.83)
Lesotho	2.07 (1.22–3.54)	26 (16–43)	880 (536–1447)	79 (72–87)	3906 (3553–4228)	0.33 (0.20–0.55)
Liberia	0.06 (0.03–0.11)	3 (1–5)	163 (96–277)	3 (3–3)	667 (624–708)	0.84 (0.48–1.47)
Madagascar	0.01 (0.01–0.02)	3 (2–5)	27 (17–43)	22 (19–24)	485 (458–508)	0.13 (0.08–0.21)
Malawi	0.45 (0.26–0.77)	57 (34–95)	3396 (2100–5497)	54 (51–56)	20766 (19558–21949)	1.06 (0.64–1.78)
Mali	0.06 (0.04–0.10)	24 (15–38)	918 (559–1511)	70 (66–73)	2023 (1870–2178)	0.35 (0.22–0.55)
Mauritania	0.01 (0.01–0.02)	0 (0–0)	11 (7–19)	0 (0–0)	0 (0–0)	NA
Mozambique	0.54 (0.28–1.06)	150 (81–277)	3684 (2069–6571)	319 (301–334)	26252 (24980–27464)	0.47 (0.25–0.87)
Namibia	2.70 (1.57–4.67)	24 (15–40)	520 (317–853)	4 (4–4)	2126 (2036–2206)	6.16 (3.74–10.22)
Niger	0.01 (0.01–0.02)	3 (2–5)	122 (72–207)	6 (6–6)	742 (679–801)	0.48 (0.28–0.83)
Nigeria	0.10 (0.06–0.16)	302 (194–469)	7663 (5014–11726)	464 (441–489)	23232 (22361–24149)	0.65 (0.42–1.02)
Rwanda	0.24 (0.13–0.44)	16 (9–28)	888 (527–1492)	7 (6–8)	2845 (2662–3016)	2.29 (1.31–4.01)
Sao Tome and Principe	0.06 (0.03–0.12)	0 (0–0)	1 (1–3)	0 (0–0)	0 (0–0)	NA
Senegal	0.03 (0.02–0.05)	3 (2–6)	189 (113–314)	9 (8–10)	708 (649–771)	0.37 (0.21–0.65)
Sierra Leone	0.27 (0.16–0.47)	25 (15–41)	693 (428–1120)	5 (4–5)	645 (613–676)	5.33 (3.18–8.89)
South Africa	3.44 (2.14–5.59)	667 (432–1037)	13411 (8865–20261)	135 (128–142)	40424 (37949–42670)	4.95 (3.20–7.69)
South Sudan	0.17 (0.09–0.32)	27 (15–47)	513 (302–870)	13 (12–13)	2071 (1839–2293)	2.08 (1.18–3.67)
Togo	0.19 (0.11–0.34)	11 (7–20)	521 (308–881)	5 (5–5)	1442 (1361–1522)	2.23 (1.30–3.81)
Uganda	0.44 (0.25–0.77)	159 (94–268)	5820 (3540–9571)	124 (118–130)	32514 (31219–33848)	1.28 (0.76–2.17)
United Republic of Tanzania	0.38 (0.21–0.71)	166 (94–292)	4625 (2794–7669)	144 (134–154)	26111 (24095–28110)	1.15 (0.65–2.03)
Zambia	0.68 (0.37–1.27)	84 (48–149)	2628 (1564–4424)	49 (47–52)	15229 (13893–16488)	1.71 (0.97–3.04)
Zimbabwe	1.70 (1.02–2.81)	185 (115–297)	6232 (3944–9862)	42 (41–44)	34579 (33243–36031)	4.40 (2.73–7.06)
Sub-Saharan Africa	0.27 (0.16–0.45)	2677 (1643–4347)	85288 (53147–136921)	2288 (2160–2413)	351089 (331150–370435)	1.17 (0.72–1.91)

Estimates with 95% confidence intervals are shown.

NA, not applicable; PTB, preterm birth.

### Trends of neonatal mortality associated with HIV-attributable preterm births in countries with the highest HIV burden in 1990–2020

**Fig. 1:**
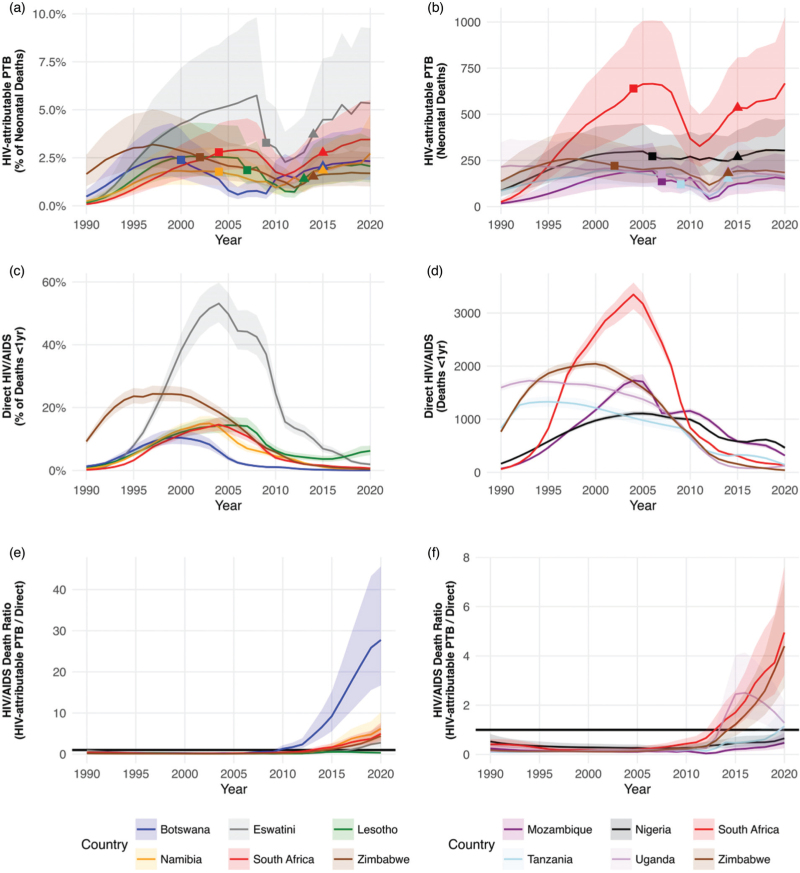
Neonatal deaths from HIV-attributable preterm birth and direct infant HIV/AIDS deaths in countries with the highest HIV burden in 1990–2020.

In the countries with the highest HIV burden, neonatal deaths associated with HIV-attributable preterm births increased during the 1990s, reaching a peak in the early 2000s, before decreasing during the 2000s, with nadirs between 2006 and 2014, and increasing again within the last decade (Fig. 1a and b). Notably, the period with the lowest proportions and numbers of HIV-attributable preterm births and associated neonatal deaths for all countries coincided with their respective periods of ZDV monotherapy administration (Fig. 1a and b). By 2020, for most countries the proportions and numbers of neonatal deaths attributable to HIV-associated preterm birth returned to levels similar to their peaks in the early 2000 s (Fig. 1a and b). Eswatini had the highest proportions of neonatal deaths due to HIV-attributable preterm births (Fig. 1a), while South Africa had the highest counts (Fig. 1b). These countries saw increases from low levels in 1990 to peaks around 2008, followed by sharp declines to nadirs in 2011, and subsequent increases leading to levels in 2020 that are similar to those in 2008 (Fig. 1a and b).

(a, c, and e) Countries with the highest proportions of neonatal deaths due to HIV-attributable preterm birth in 2020. (b, d, and f) Countries with the highest numbers of neonatal deaths due to HIV-attributable preterm birth in 2020. (a) Proportions of neonatal deaths due to HIV-attributable preterm birth in Botswana, Eswatini, Lesotho, Namibia, South Africa, and Zimbabwe, 1990–2020. The earliest and latest years of antenatal administration of zidovudine monotherapy for each country are indicated with squares and triangles, respectively. (b) Numbers of neonatal deaths due to HIV-attributable preterm birth in Mozambique, Nigeria, South Africa, Tanzania, Uganda, and Zimbabwe, 1990–2020. The earliest and latest years of antenatal administration of zidovudine monotherapy for each country are indicated with squares and triangles, respectively. (c) Direct HIV/AIDS deaths among infants <1 year old as a proportion of all deaths <1 year in Botswana, Eswatini, Lesotho, Namibia, South Africa, and Zimbabwe, 1990–2020. (d) Numbers of direct HIV/AIDS deaths among infants <1 year old in Mozambique, Nigeria, South Africa, Tanzania, Uganda, and Zimbabwe, 1990–2020. (e) Ratio of neonatal deaths due to HIV-attributable preterm birth and direct HIV/AIDS deaths <1 year in Botswana, Eswatini, Lesotho, Namibia, South Africa, and Zimbabwe, 1990–2020. (f) Ratio of neonatal deaths due to HIV-attributable preterm birth and direct HIV/AIDS deaths <1 year in Mozambique, Nigeria, South Africa, Tanzania, Uganda, and Zimbabwe, 1990–2020. PTB, preterm birth.

### Trends of direct HIV/AIDS infant deaths and neonatal deaths from HIV-attributable preterm birth in countries with the highest HIV burden in 1990–2020

In the period 1990–2020, there were an estimated 351 089 (331 150–370 435) infant deaths <1 year directly due to HIV/AIDS in sub-Saharan Africa (Table 1). In the countries with the highest HIV burden, the proportions and numbers of direct HIV/AIDS infant deaths <1 year increased during the 1990 s, reaching a peak between 1995–2005, before decreasing to the levels seen in 2020 (Fig, 1c and d). When neonatal deaths due to HIV-attributable preterm birth were divided by direct HIV/AIDS infant deaths <1 year (presented as a ratio) it became apparent that in the last decade neonatal deaths due to HIV-attributable preterm birth exceeded direct HIV/AIDS infant deaths <1 year (ratio >1) and continue to increase in most countries (Fig. 1e and f). By 2020, the ratio of neonatal deaths due to HIV-attributable preterm birth over direct HIV/AIDS infant deaths <1 year was 27.61 (16.52–45.83) in Botswana, 6.16 (3.74–10.22) in Namibia, 4.95 (3.20–7.69) in South Africa, and 4.40 (2.73–7.06) in Zimbabwe (Table 1).

## Discussion

To our knowledge, this is the first study to provide country-level estimates of neonatal mortality attributable to preterm births associated with maternal HIV infection in sub-Saharan Africa in 1990–2020. We found that during 1990–2020 there were an estimated 85 288 (53 147–136 921) neonatal deaths due to HIV-attributable preterm births in sub-Saharan Africa. The proportion and number of neonatal deaths due to HIV-attributable preterm births in sub-Saharan Africa increased during the 1990s, before decreasing in the 2000s, reaching a nadir around 2012, and increasing again within the last decade to levels in 2020 that are similar to those in the early 2000 s. Maternal HIV made the largest contribution to the burden of preterm birth and associated neonatal mortality in high HIV prevalence countries in southern sub-Saharan Africa. The proportions and numbers of infant deaths directly due to HIV/AIDS have consistently declined since their peak in the early 2000 s. Taken together, our analysis shows that neonatal deaths due to HIV-attributable preterm birth are increasing and exceed direct infant deaths due to HIV/AIDS in sub-Saharan Africa in 2020.

Over time, changing WHO recommendations of ART regimens for use during pregnancy have influenced HIV-attributable preterm birth and associated neonatal deaths. Historically, pregnant WWH who received ZDV monotherapy had a risk of preterm birth comparable to HIV-negative women [9]. From 2013, many countries shifted to combination ART, which is associated with an increased risk of preterm birth, relative to women without HIV [9]. Since 2015, the WHO has recommended that all people living with HIV initiate lifelong combination ART as soon as possible after diagnosis [7], which increased preconception ART and the risk of preterm birth [10,15,16]. These trends highlight how changes in HIV treatment policies have driven preterm birth outcomes [12].

This study has several strengths. To our knowledge, it provides the first country-level estimates of neonatal mortality associated with HIV-attributable preterm birth in sub-Saharan Africa in 1990–2020. We leverage several large international data sets, including results of a large meta-analysis of 34 cohort studies assessing the associations between WWH receiving different antiretroviral regimens with preterm birth, including 399 558 women in 14 countries in sub-Saharan Africa. Moreover, we integrate these meta-analysis results with estimates from the GBD Study 2021 and UNAIDS data to generate unique estimates of neonatal mortality associated with HIV-attributable preterm birth.

This study also has some limitations. Lacking direct estimates of neonatal deaths associated with maternal HIV and ART, we used the most important contributor, preterm births, as a proxy and assumed that the mortality rate for HIV-associated and non-HIV-related preterm births were consistent. We could not estimate the effect of other HIV-related adverse perinatal outcomes that lead to mortality, such as stillbirth, low birthweight, and small for gestational age [8–10], so our model likely underestimates the overall impact of maternal HIV. Finally, we could not account for the different antiretroviral drugs that may have been part of the triple drug ART regimens used in different countries, despite evidence that different classes of antiretroviral drugs are associated with different comparative risks of adverse perinatal outcomes [17–19].

Reducing preterm births and neonatal deaths from HIV-attributable preterm birth will require a multifaceted approach. Although preconception ART has clear benefits for maternal health and prevention of vertical transmission of HIV, regimens used in pregnancy can be further optimized to reduce the incidence of preterm births and other adverse perinatal outcomes. Primary prevention of HIV infection should be scaled up, including oral pre-exposure prophylaxis and long-acting injectables cabotegravir and lenacapavir, with improved collection of safety data in pregnancy [20]. Further studies should aim to develop further preventive and therapeutic interventions to reduce HIV-attributable preterm birth and associated neonatal deaths, particularly in countries with a high HIV prevalence. Beyond HIV-specific measures, evidence-based interventions to reduce the incidence of preterm birth and improve outcomes of premature babies is crucial to achieve the Sustainable Development Goal 3 target of ending preventable deaths of newborns and children under age five by 2030 [21].

## Acknowledgements

Author contributions: N.N. cleaned and analyzed the data, developed the figures and tables, interpreted the data and wrote the first draft of the manuscript. JH conceived and designed the study, designed the analysis, figures and tables, interpreted the data and wrote the manuscript. All authors read and approved the final version of the manuscript.

Funding: None.

### Conflicts of interest

There are no conflicts of interest.
